# Deformation-Induced Crystal Growth or Redissolution, and Crystal-Induced Strengthening or Ductilization in Metallic Glasses Containing Nanocrystals

**DOI:** 10.3390/ma17112567

**Published:** 2024-05-27

**Authors:** Tittaya Thaiyanurak, Saowaluk Soonthornkit, Olivia Gordon, Zhenxing Feng, Donghua Xu

**Affiliations:** 1 Materials Science Program, Oregon State University, Corvallis, OR 97331, USA; 2 School of Mechanical, Industrial and Manufacturing Engineering, Oregon State University, Corvallis, OR 97331, USA; 3 School of Chemical, Biological and Environmental Engineering, Oregon State University, Corvallis, OR 97331, USA; 4 Engineering Program, Clackamas Community College, Oregon City, OR 97045, USA

**Keywords:** metallic glasses, crystals, deformation, shear bands, strength and ductility

## Abstract

It is generally known that the incorporation of crystals in the glass matrix can enhance the ductility of metallic glasses (MGs), at the expense of reduced strength, and that the deformation of MGs, particularly during shear banding, can induce crystal formation/growth. Here, we show that these known trends for the interplay between crystals and deformation of MGs may hold true or become *inverted* depending on the size of the crystals relative to the shear bands. We performed molecular dynamics simulations of tensile tests on nanocrystal-bearing MGs. When the crystals are relatively small, they bolster the strength rather than the ductility of MGs, and the crystals within a shear band undergo redissolution as the shear band propagates. In contrast, larger crystals tend to enhance ductility at the cost of strength, and the crystal volume fraction increases during deformation. These insights offer a more comprehensive understanding of the intricate relationship between deformation and crystals/crystallization in MGs, useful for fine-tuning the structure and mechanical properties of both MGs and MG–crystal composites.

## 1. Introduction

Metallic glasses (MGs), also known as amorphous metals or alloys, are a highly innovative family of metallic materials. They differ drastically from conventional metals and alloys (including high-entropy alloys) in both structure and properties. MGs possess an overall disordered atomic structure, without crystal gains and crystal defects (e.g., dislocations and grain boundaries) that are commonly present in the conventional metals and alloys. Owing to their unique atomic structure, MGs exhibit very high strength, hardness, elastic strain limit and wear and corrosion resistance, far exceeding their crystalline counterparts [1,2,3,4,5,6].

Nevertheless, MGs are still facing a few hurdles before they can be widely used in engineering applications. For example, their compositions, especially those with relatively good glass-forming ability (GFA), are not yet engineering friendly, often relying upon toxic, precious or rare-earth elements [6,7,8,9,10,11,12,13,14]. Several recent discoveries [15,16,17] of nontoxic, precious metal and rare-earth free MGs with an exceptional GFA have addressed this problem to some extent, although continued effort is evidently required. Another hurdle that MGs are facing is their limited ductility (or plasticity) [18,19,20,21]. Although possessing superior strength and fairly high toughness, most MGs exhibit no or a rather limited plastic strain when they finally fracture, especially under a tensile loading condition. Introducing crystal phases, either extrinsically added or intrinsically formed during melt casting, into the MG matrix has been proposed as a strategy to improve the ductility of MGs [18,22,23,24,25,26,27]. Many experimental studies [22,23,24,25,26,27] have reported the success of this ductilization strategy, although at the expense of reduced strength. The ductilization effect has been explained mainly on the basis of hypothesized interactions between the crystal phases and shear bands in the MG matrix.

Shear bands are thin layers of atoms in MGs that experience significantly more shear strain than the other atoms. They are the main carrier of plasticity near and after yielding. Shear bands are also directly related to the final fracture of an MG, which can be caused by either the rapid propagation of a single dominant shear band across the sample width or by the transformation of a shear band into a crack that propagates rapidly. Crystal phases embedded in an MG matrix are generally believed to interrupt (slowing down or redirecting) the propagation of shear bands and their transformation into cracks in the MG matrix, hence delaying the fracture and improving the ductility.

Related to this but from a different perspective, (nano-)crystals have been reported in a fair number of studies to form inside propagating shear bands based on electron micrographs obtained after the deformation and/or fracture of MGs [28,29,30,31,32]. This has been interpreted as the result of increased atomic volume (shear dilation), enhanced atomic mobility and/or increased temperature in the shear bands.

These two trends, namely crystals in the glass matrix ductilizing MGs (at the expense of reduced strength) and deformation (particularly shear banding) promoting crystal formation/growth, represent arguably the most intriguing view of the interplay between the deformation of MGs and crystals inside the glass matrix. However, to what extent these trends are valid is still in question. For example, a brittle crystal phase is generally not expected to be able to ductilize an MG, and shear bands have not been found to trigger crystal formation/growth in many MGs. Meanwhile, the fundamental mechanisms enabling or disabling these trends are still not well understood.

Here, we study the effects of the size of crystals contained in an MG on their interplay with the deformation (particularly shear banding) of the MG, from both perspectives of ductilization and crystal formation/growth. We performed molecular dynamics (MD) simulations of tensile tests on single-element tantalum (Ta) MGs containing different-sized, simple body-centered cubic (BCC)-structured nanocrystals that were spontaneously formed during prior thermal devitrification. We show that the aforementioned trends, i.e., crystals ductilizing MGs and shear bands promoting crystal formation/growth, may hold true or become *inverted* depending on the size of the crystals relative to the shear bands. This provides new insights into the intricate relationship between deformation and crystals/crystallization in MGs.

## 2. Simulation Methods

All the MD simulations in this study were performed using the open-source LAMMPS (Large-Scale Atomic/Molecular Massively Parallel Simulator) code (version: 3 Mar 2020) [33] that was developed and distributed by the Sandia National Laboratory. Elemental tantalum (Ta) was chosen to be the subject material because Ta has an optimal balance of chemical simplicity, glass-forming ability, thermal stability in the solid glass regime, and tendency to crystallize (BCC) in the supercooled liquid regime [34,35,36]. The EAM (Embedded Atom Method) potential [36] developed specifically for Ta on the basis of both experimental and quantum mechanical data was employed in all the MD simulations.

A perfect BCC crystal of Ta with 871,200 atoms (110, 18 and 220 unit cells in x, y and z directions, respectively) was first melted and equilibrated at 4000 K under the NPT (controlled particle number, N; pressure, P; and temperature, T) ensemble and all periodic boundary conditions. Then, the molten Ta was cooled down to 300 K at a cooling rate of 1 K/ps, also under the NPT ensemble and all periodic boundary conditions. This resulted in a fully glassy sample with a rectangular (strip) shape and dimensions of ~37 × 6 × 74 nm. Then, the sample was trimmed in the x-direction (only), reducing the x-dimension to ~18 nm, to achieve an aspect ratio of ~3:1:12, which is more typical of tensile strip specimens. The periodic boundary condition was turned off in the x and y directions. The sample was saved as Sample S1, i.e., the fully glassy sample, for later tensile testing.

Two replicated copies of S1 were annealed at 1400 K, one for 1 ns and the other for 2.2 ns, in order to form nanocrystals of different sizes (within a remaining glassy matrix). These two samples were then cooled down to 300 K at a cooling rate of 10 K/ps and saved as Sample S2 and Sample S3, respectively.

The fully glassy Sample S1 and the partially devitrified Samples S2 and S3 were all relaxed at 300 K for 50 ps and then subjected to a uniaxial tensile loading along the z-direction at a strain rate of 10^−4^/ps until fracture. During the tensile tests, the Nose–Hoover thermostat was used to maintain the sample temperature at 300 K.

Post-simulation visualization, atomic structure and atomic strain analyses were conducted using the Ovito (Open VIsualization TOol) program (version: 3.10.2) [37]. More specifically, the radial distribution function (RDF) was used to evaluate the overall structure (ordered or disordered) of each sample prior to the tensile test. The polyhedral template matching (PTM) algorithm (with the root mean square deviation (RMSD) parameter set to 0.12) in Ovito was used to identify the BCC atoms (i.e., those atoms possessing a local BCC atomic environment) and their clusters (i.e., BCC crystals or grains) before and during the tensile test. The atomic shear strain (with respect to the initial atomic configuration at the start of each tensile test) was calculated by Ovito and was used to visualize shear transformation zones (STZs, groups of atoms that have undergone more shearing than the others, which appear earlier than shear bands) and shear bands and to analyze the interaction between shear bands and crystals during continued deformation. Ovito was also used to measure the actual sample volume (through the “construct surface mesh” function) at varied strains. The actual sample volume was used to correct the nominal stress (“pzz”) evaluated by LAMMPS, which was based on the simulation-box volume.

## 3. Results and Discussion

**Figure 1:**
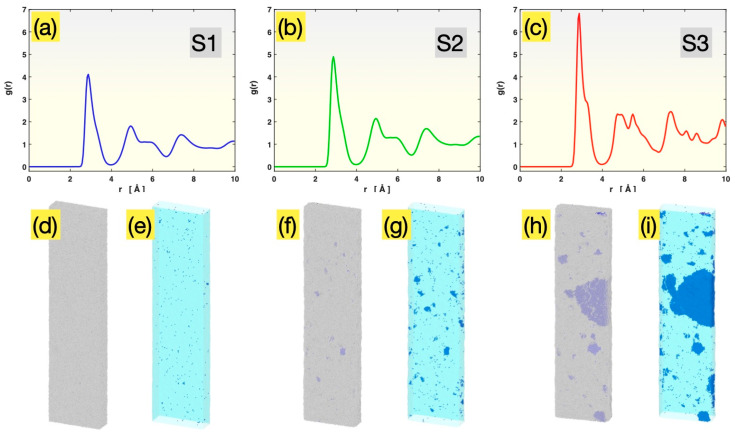
Initial structures of Sample S1 (**a**,**d**,**e**), Sample S2 (**b**,**f**,**g**) and Sample S3 (**c**,**h**,**i**). Panels (**a**–**c**): the radial distribution functions. Panels (**d**,**f**,**h**): the overall external views of all atoms (blue, BCC atoms; gray, disordered atoms). Panels (**e**,**g**,**i**): the views of BCC atoms only (with semitransparent constructed sample surface).

Figure 1 presents the initial atomic structure information for Samples S1, S2 and S3, after relaxation at 300 K, but before the tensile test. As seen in Figure 1a, Sample S1 possesses an RDF, $g\left(r\right)$ versus *r*, that consists of broad peaks representing different (1st, 2nd, …) coordination shells, with splittings on the second peak, as is typical of metallic glasses. Defined to be the atomic number density ($\rho_{r}$) within a radial distance of *r ~ r* + *dr* from an average atom, normalized by the overall atomic number density ($\rho_{tot}$) in the material, i.e., $g\left(r\right)=\frac{\rho (r)}{\rho_{tot}}=\frac{dN(r)}{\rho_{tot}4\pi r^{2}dr}$, the RDF represents the relative probability of finding other atoms at varied interatomic distances which depends on the atomic structure of the subject material. For a crystalline material with well-developed ordering, the atoms are located at only specific interatomic distances, and hence its RDF consists of a series of sharp peaks (spikes). For a glass, the atoms are largely disordered, and the RDF peaks are significantly broadened, similar to those in Figure 1a.

Unlike the RDF evaluation, which involves averaging over all atoms, the PTM analysis identifies the local structure around each individual atom (by comparing its neighbors’ spatial arrangement with templates for different structures, here BCC). For S1, only 0.08% of all atoms were identified by the PTM analysis to have a local BCC environment. These atoms are colored blue in Figure 1d,e (as well as in Figure 1f–i for the other samples). They are barely visible in Figure 1d, with the other 99.92% of atoms (colored gray) shown together. When those non-BCC atoms are made invisible, as in Figure 1e, the 0.08% BCC atoms are seen to be randomly distributed in the sample and mostly isolated from each other, with only a few extremely small clusters (<10 atoms each). These BCC atoms are the result of thermodynamic fluctuation in an essentially fully glassy sample and are not real crystals.

Sample S2, having been annealed at 1400 K for 1 ns, still possesses an RDF (Figure 1b) very similar to that of Sample S1 (Figure 1a). However, the height of the peaks within the 0 to 10 Å range is increased, indicating that the probability of finding atoms within this range is enhanced relative to the probability at farther distances (beyond 10 Å). This subtle change in the RDF is attributable to the formation of small crystals during the 1 ns annealing, although the remaining glassy matrix still dominates the averaging involved in the RDF evaluation. The presence of small crystals in S2 becomes more evident upon the PTM analysis, as shown in Figure 1f,g. A substantial number of crystal grains with dimensions in the range of 1 to 3 nm, containing a few tens to ~200 atoms each, are clearly visible. The total fraction of the BCC atoms in Sample S2 is 0.5% prior to the tensile test.

Sample S3, having been annealed at 1400 K for 2.2 ns, possesses an RDF, as shown in Figure 1c, that displays mixed glass and crystal characteristics, with sharp (crystalline) peaks appearing on top of a broad (glassy) pattern. The PTM analysis, as presented in Figure 1h,i, further shows the presence of many crystal grains with different sizes, ranging from ~1 nm to ~20 nm (in the longest dimension). The total fraction of the BCC atoms in Sample S3 was 13.4% prior to the tensile test.

Given their initial atomic structures discussed above, these three samples are representative of three different scenarios: Sample S1, fully glassy material; Sample S2, a mostly glassy material with small crystals; and Sample S3, a largely glassy material containing some big crystals.

**Figure 2:**
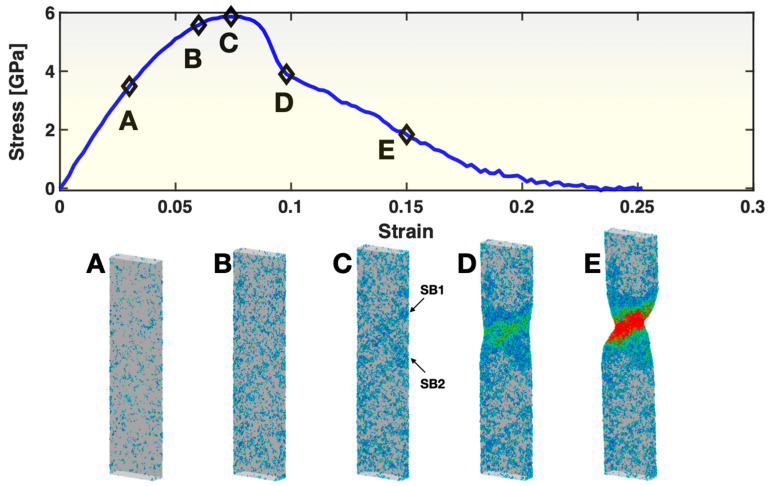
Stress–strain curve (**upper panel**) and views of atoms with relatively large (≥0.3) atomic shear strain (**lower panel**) in Sample S1. In the lower panel, the color represents the atomic shear strain (blue being the lowest, red the highest). The five frames in the lower panel correspond to the five states (**A**–**E**) marked on the stress–strain curve at a strain level of 0.03, 0.06, 0.074, 0.098 and 0.15, respectively. For frame C, two arrows point out the locations/orientations of two shear bands (SB1 and SB2) developed soon after this state.

The upper panel of Figure 2 presents the stress–strain curve for Sample S1. It displays the typical characteristics of MGs commonly seen in MD simulated tensile tests. The curve starts with a relatively straight segment, then bends, reaches a maximum in stress and then exhibits a sudden drop of stress, after which some additional deformation (strain) takes place, and the stress continues to decrease until fracture (zero stress). To illustrate the atomic behaviors at the different stages, five states, A, B, C, D and E, are selected from the stress–strain curve for atomistic visualization. In state A, where the deformation is mostly elastic, there are already small groups of atoms, i.e., STZs, that have undergone a relatively high atomic shear strain (≥0.3), as seen in the first frame of the lower panel of Figure 2. These STZs are nearly uniformly distributed on the sample surface (we previously reported the surface condensation of STZs in this deformation regime [38]), but they are not interconnected with each other. In state B, the population of STZs (both on the surface and inside the sample) has increased to such a degree that they start to show some correlation/patterning in certain directions/planes. In state C, with an even higher population of STZs, the spatial correlation among some of the STZs has reached a length scale close to the sample dimensions, causing the formation and activation/operation of two shear bands (SB1 and SB2, as marked by the two arrows in the figure) and the yielding of the sample (signified by the rapid drop of stress) soon after state C. By state D, one of the shear bands (SB1) has become dominant. From then on, the sample deforms by the propagation of SB1, through state E, until fracture.

**Figure 3:**
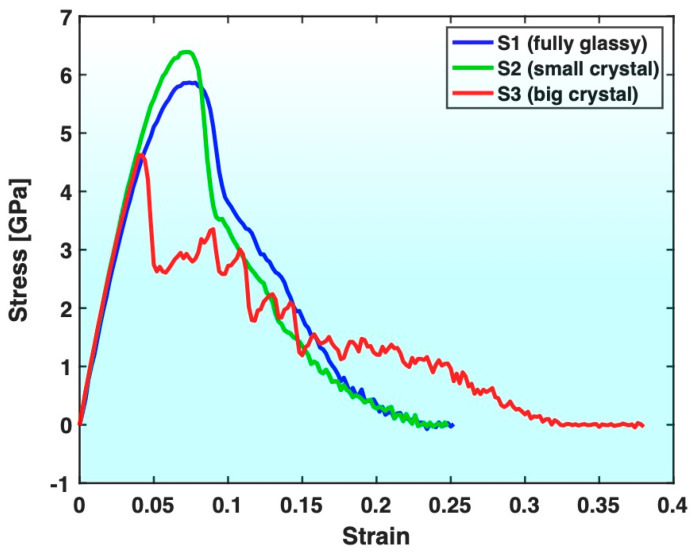
Comparison of stress–strain curves of Samples S1, S2 and S3.

Figure 3 presents the stress–strain curves of all three samples. Compared with Sample S1 (fully glassy), Sample S3 (containing big crystals) exhibits a lower yield strength, earlier yielding and more gradual approach of stress to zero after yielding, together with a notably larger fracture strain. Evidently, Sample S3 was ductilized by the crystals contained in its glassy matrix, at the price of lowered strength, which is consistent with the known trend of crystals ductilizing MGs. On the other hand, Sample S2 (containing small crystals) exhibits a higher yield strength and a more rapid drop in stress upon and after yielding than Sample S1 (although the final fracture strain is not much different). This indicates that the small crystals contained in the glassy matrix of S2 have strengthened the sample with a slight loss of ductility, which is opposite to the known trend of crystals ductilizing MGs and lowering their strength.

**Figure 4:**
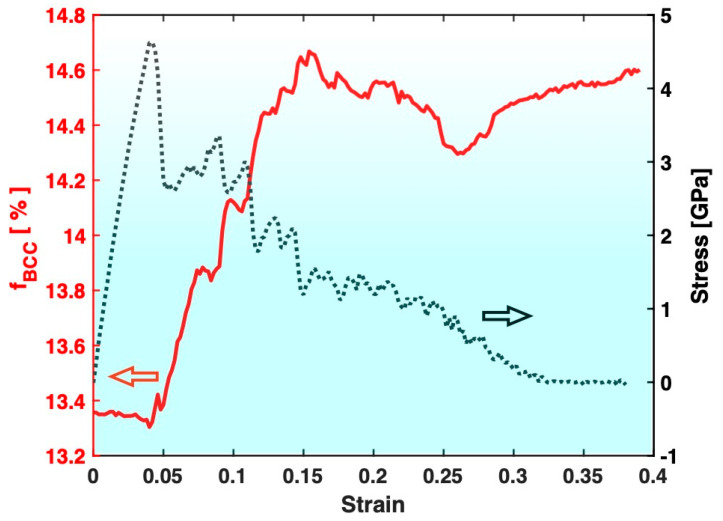
Variation in the fraction of BCC atoms (f_BCC_, red) during the tensile test of Sample S3. The stress–strain curve (black) is also plotted for reference.

**Figure 5:**
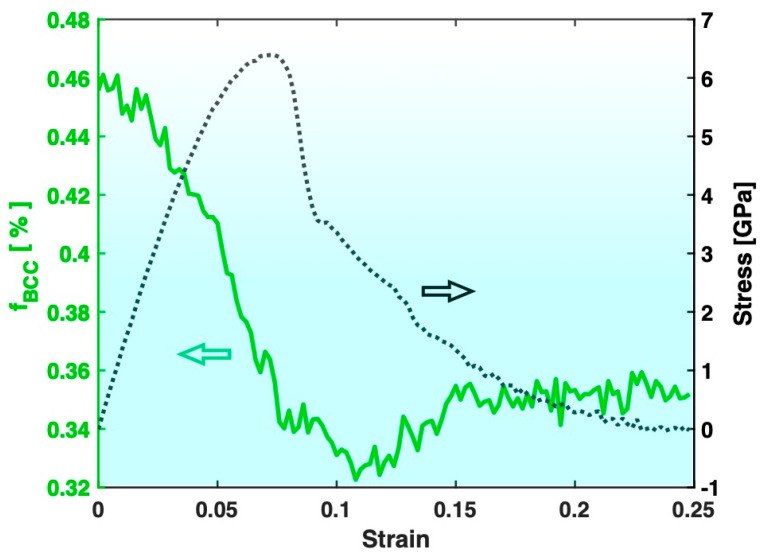
Variation in the fraction of BCC atoms (f_BCC_, green) during the tensile test of Sample S2. The stress–strain curve (black) is also plotted for reference.

Figure 4 presents the variation in the fraction of the BCC atoms in Sample S3 (containing big crystals) during the tensile test, together with the stress–strain curve. The most evident feature is that the fraction of the BCC atoms in Sample S3 increases significantly after the tensile test, which is consistent with the known trend of deformation promoting crystal formation/growth. On the other hand, as shown in Figure 5, the fraction of the BCC atoms in Sample S2 (containing small crystals) experiences a significant drop after the tensile test. This indicates that the deformation in Sample S2 has redissolved some of the pre-existing crystals, which is contrary to the known trend of deformation promoting crystal formation/growth.

**Figure 6:**
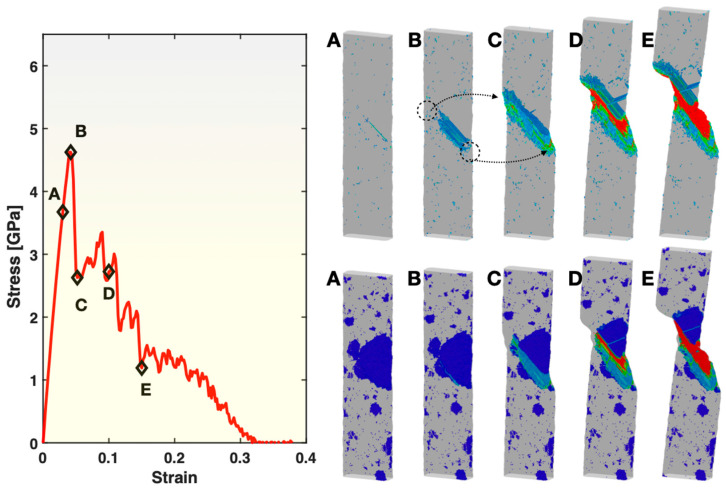
Stress–strain curve (**left panel**), views of atoms with relatively large (≥0.3) atomic shear strain (**top right panel**), and views of BCC atoms (**bottom right panel**) in Sample S3. In both of the right panels, the color represents the atomic shear strain (blue being the lowest, red the highest). The markings (circles and arrows) in the top right panel are explained in the main text. The five frames in the top right and the bottom right panels correspond to the five states (**A**–**E**) marked on the stress–strain curve, at a strain level of 0.03, 0.042, 0.052, 0.1 and 0.15, respectively.

To understand the different trends displayed in Figure 3, Figure 4 and Figure 5 and the atomic mechanisms involved, atomistic visualization was performed for Samples S2 and S3 at varied stages of deformation. Presented in the top right panel of Figure 6 are the views of the atoms with a relatively high (≥0.3) atomic shear strain in Sample S3 for the five states marked on the stress–strain curve in the left panel of the same figure. In state A (mostly elastic deformation), a rather small number of atoms have reached above the 0.3 atomic shear strain threshold (hence visible in the figure), some of which are scattered in the glass matrix and some along the edge of the grain boundary between two crystals (see state A in the bottom right panel for corresponding crystal locations). In state B (just before yielding), the number of atoms in the glassy matrix reaching above 0.3 atomic shear strain has experienced a slight increase but remains relatively low. However, the atoms in nearly the whole grain boundary between the two crystals mentioned above have now experienced ≥0.3 atomic shear strain. Shortly after this, shear banding takes place in the glassy matrix on the two sides (circled out in the figure) of the grain boundary, causing the sample to yield and start to undergo significant shearing and shape change. The yielding here takes place at an overall tensile strain of ~0.042, which is notably earlier than the yielding in the fully glassy Sample S1 (at 0.074 overall tensile strain). This is attributable to the stress concentration on the two sides of the shearing/sliding grain boundary between the two crystals. In the subsequent (post-yielding) deformation of Sample S3, as represented by states C, D and E, the two sides of the operating shear band are blocked by the crystals in between. Consequently, the shear band cannot cut through the width of the sample as easily as in the fully glassy Sample S1 (shown in Figure 2). Instead, it has to operate together with grain boundary sliding and grain shearing/elongation (visible in the bottom right panel of Figure 6), which results in a bigger plastic zone and larger overall fracture strain than in Sample S1.

The bottom right panel of Figure 6 presents the views of all BCC atoms and, hence, the crystal grains present in the glassy matrix (invisible), corresponding to the same five states of Sample S3 as in the top right panel of the figure. It shows the location of the grain boundary shearing/sliding that is responsible for triggering shear banding of the glass matrix and the yielding of the sample, as well as the morphological changes to the crystal grains during the post-yielding deformation, as discussed above. Moreover, these images show that the crystal grains outside the plastic zone do not experience any noticeable changes to their shapes and sizes. However, the multiple crystal grains within the plastic zone that possess relatively big sizes undergo merging and re-organization, in addition to the shearing and shape change. The merging and re-organization of the grains under deformation over states C, D and E have converted some of the atoms originally in the glassy matrix into part of the grains, resulting in the increment of the overall fraction of BCC atoms shown earlier in Figure 4.

**Figure 7:**
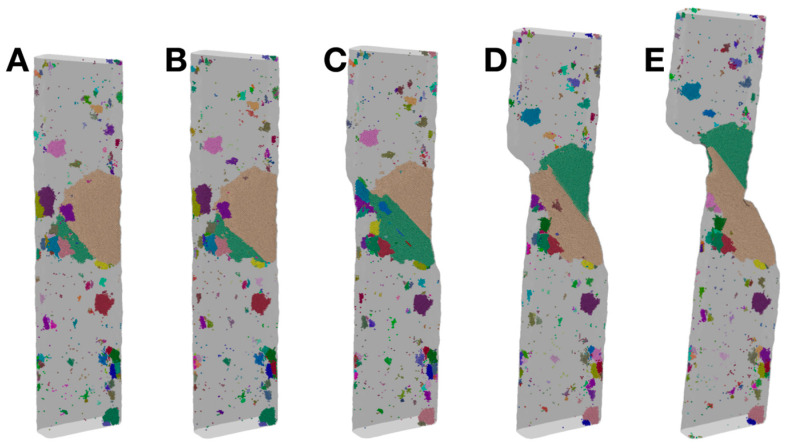
Views of crystal grains in Sample S3, corresponding to the same five states (**A**–**E**), as shown in Figure 6, at a strain level of 0.03, 0.042, 0.052, 0.1 and 0.15, respectively. Note that the colors are used to distinguish the grains within each state, but the colors are not directly correlated across the different states (due to the changes to some grains and the remapping of the colors).

Figure 7 presents another way of visualizing the crystals during the deformation of Sample S3, for the same five states (strain levels) as in Figure 6. It uses different colors to distinguish the many grains within each state (note that the colors are not directly correlated across different states due to the changes to some grains and the remapping of the colors). Grains neighboring each other and the boundaries between them are recognized more easily in this visualization. Figure 7 again shows that the big crystal grains within the plastic zone experience significant changes in their shapes, relative sizes and locations, as a result of the deformation, while those crystal grains far from the plastic zone are basically not affected.

To better understand the increase in the total crystalline fraction, i.e., the fraction of the BCC atoms, presented in Figure 4, the local temperature during deformation was analyzed inside Ovito, using the atomic kinetic energy data from LAMMPS. With the Nose–Hoover thermostat controlling the overall temperature of the sample during the MD simulation, the local temperature near the plastic zone was raised to no more than 500 K in Sample S3 throughout the process, which is far below the annealing temperature (1400 K) used to form the crystals in the sample preparation stage. This suggests that the local heating is not the cause of the increment of the crystal fraction. Rather, the deformation itself is responsible for the growth (as measured by the number fraction of BCC atoms) of the big crystals within the plastic zone.

**Figure 8:**
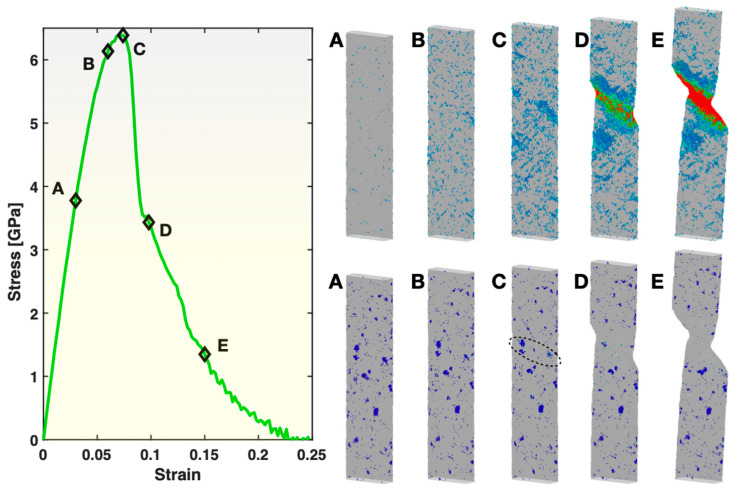
Stress–strain curve (**left panel**), views of atoms with relatively large (≥0.3) atomic shear strain (**top right panel**) and views of BCC atoms (**bottom right panel**) in Sample S2. In both of the right panels, the color represents the atomic shear strain (blue being the lowest, red the highest). The five frames in the top right and the bottom right panels correspond to the five states (**A**–**E**) marked on the stress–strain curve at a strain level of 0.03, 0.06, 0.074, 0.098 and 0.15, respectively.

Figure 8 presents the views of the large-shear-strain (≥0.3) atoms (top right panel) and the BCC atoms (bottom right panel) for five states during the deformation of Sample S2 (containing small crystals). These five states correspond to the same five levels of overall tensile strain as used in Figure 2 for the fully glassy Sample S1. In the pre-yielding states A and B, the spatial distribution of the atoms/STZs with ≥0.3 atomic shear strain in Sample S2 is similar to that in Sample S1 at the same levels of overall tensile strain. However, their population is much lower in Sample S2 here than in Sample S1. This indicates that the glass matrix has been strengthened (more elastic at a given tensile strain) by the existing small crystals. In the state C (just before yielding), the STZs exhibit clear spatial correlations along certain directions and planes, thus quickly triggering the formation and activation of one single shear band (see state D) and, hence, yielding of the sample. This is similar to Sample S1, but with a noticeable difference that the spatial correlations among the STZs here in Sample S2 are more localized (shorter range). After yielding, the single active shear band propagates through the width of the sample, as seen in the states D and E, causing the overall stress to drop towards zero, until the final fracture of the sample. The faster stress drop towards zero in Sample S2 than in Sample S1 upon and after yielding, as exhibited in Figure 3, can now be understood as the result of the more localized spatial correlations of STZs and the single operating shear band at the time of yielding in Sample S2.

As evident in the bottom right panel of Figure 8, most of the small crystals in Sample S2 do not experience noticeable changes during the tensile test. However, those located within or near the pathway of the active shear band, as circled out in the figure, are eliminated (redissolved) by the shear band. The temperature analysis within Ovito revealed that the local temperature within the plastic zone reached ~600 K at maximum, slightly higher than in Sample S3 but still far below the original annealing temperature of 1400 K used to form the crystals in the sample-preparation stage. Hence, the deformation itself, instead of the local heating, is responsible for the redissolution of the small crystals. Indeed, local heating below 1400 K, even if significant enough, would only promote the growth of the crystals, as the thermodynamic driving force for crystallization is stronger at lower temperatures.

The striking differences between Sample S2 and Sample S3, both containing crystals in the glassy matrix, are evidently related to the size of the crystals—with respect to the shear bands. In Sample S2, the crystals are very small. They suppress the formation of STZs, making the material behave stronger prior to yielding. Yet, the small crystals do not significantly alter the yielding mechanism, which still requires the spontaneous formation of shear bands in the glassy matrix, the same as in the fully glassy Sample S1. This results in the apparent strengthening of the sample. In contrast, in Sample S3, the crystals are relatively big, and the crystal grain boundaries start shearing/sliding at relatively low stress, causing stress concentration and earlier shear banding in the neighboring glass matrix. This makes yielding occur sooner than in Sample S2 (and Sample S1), lowering the apparent strength of the material. After yielding, the big crystals in the pathway of the propagating shear band in Sample S3 have to be continuously sheared/deformed, preventing the rapid propagation of the shear band normally seen in a fully glassy sample, leading to apparent ductilization. In Sample S2, the small crystals cannot prevent the rapid propagation of the active shear band. The post-yielding decline of stress is even a bit faster in Sample S2 than in the fully glassy Sample S1 due to the more localized STZs and embryonic shear bands in the small-crystal-bearing sample. In terms of deformation effects on crystal growth or redissolution, the big crystals in Sample 3, located in the pathway of the active shear band, “absorb” more atoms from the nearby glassy matrix as they are reconstructed and reshaped by deformation, leading to a noticeable increase in the crystalline fraction, i.e., effective crystal growth. The small crystals in Sample 2 in the pathway of the propagating shear band get “destroyed” and converted back to a disordered glassy state by the shear band, leading to a noticeable decrease in the crystalline fraction, i.e., effective crystal redissolution.

## 4. Conclusions

We studied the interplay between deformation and crystals in a metallic glass matrix using molecular dynamics simulations of tensile tests of partially devitrified Ta metallic glass containing BCC crystals of different sizes. Our results show that when the crystals are big (relative to the shear band), they can vastly alter the yielding and the post-yielding plastic deformation mechanisms of the base metallic glass, leading to apparent ductilization and lowered strength. Meanwhile, the post-yielding deformation leads to an increased crystal fraction or, effectively, crystal growth in the material. In contrast, when the crystals are small, they can suppress the formation of shear transformation zones and increase the localization of shear transformation zones and embryonic shear bands without significantly altering the yielding and the post-yielding deformation mechanisms, leading to apparent strengthening and mild loss of ductility. The small crystals encountered by the propagating shear band after yielding are redissolved into the glassy matrix. These results provide a more complete picture of the interplay between deformation and crystals/crystallization in MGs, which could help better design the structure and mechanical properties of both metallic glasses and metallic glass–crystal composites.

## Data Availability

The data presented in this study are available on request from the corresponding author. The data are not publicly available due to the large volume, which requires significant storage resources, and the complexity, which necessitates detailed instructions for specific use.
